# Whole-Genome Sequencing of a Potential Ester-Synthesizing Bacterium Isolated from Fermented Golden Pomfret and Identification of Its Lipase Encoding Genes

**DOI:** 10.3390/foods11131954

**Published:** 2022-06-30

**Authors:** Huifang Wang, Yanyan Wu, Yueqi Wang

**Affiliations:** 1 College of Food Science and Engineering, Ocean University of China, Qingdao 266000, China; wanghfsky@163.com; 2 Key Laboratory of Aquatic Product Processing, Ministry of Agriculture and Rural Affairs, National R&D Center for Aquatic Product Processing, South China Sea Fisheries Research Institute, Chinese Academy of Fishery Sciences, Guangzhou 510300, China; 3 Co-Innovation Center of Jiangsu Marine Bio-Industry Technology, Jiangsu Ocean University, Lianyungang 222005, China; 4 Collaborative Innovation Center of Seafood Deep Processing, Dalian Polytechnic University, Dalian 116034, China

**Keywords:** *Acinetobacter venetianus*, whole genome, ester synthesis, traditional fermented golden pomfret

## Abstract

Microbial ester synthases are regarded as valuable catalysts in the food industry. Here, one strain of *Acinetobacter venetianus* with ester synthase-production capacity, SCSMX-3, was isolated from traditional fermented golden pomfret. It exhibited good growth in mesophilic, low salt, and slightly alkaline environments. The ester synthase produced by SCSMX-3 displayed maximum activity at pH 8.0 and 35 °C. Genome sequencing revealed that the strain contains one circular chromosome of 336313 bp and two circular plasmids (plasmid A-14424 bp and plasmid B-11249 bp). Six CRISPR structures enhance the genomic stability of SCSMX-3 and provide the opportunity to create new functional strains. Gene function analysis indicated that SCSMX-3 produces the necessary enzymes for survival under different conditions and for flavor substance synthesis. Furthermore, 49 genes encoding enzymes associated with lipid metabolism, including three triacylglycerol lipases and two esterases, were identified through the NCBI Non-Redundant Protein Database. The lipase encoded by gene0302 belongs to the GX group and the abH15.02 (*Burkholderia cepacia* lipase) homolog of the abH15 superfamily. Our results shed light on the genomic diversity of and lipid metabolism in *A. venetianus* isolated from fermented golden pomfret, laying a foundation for the exploration of new ester synthases to improve the flavor of fermented fish products.

## 1. Introduction

Natural fermentation is a traditional food processing method for prolonging the storage life of food while improving its sensory and functional properties [1]. Pomfrets belong to the family Bramidae and include golden pomfret, silver pomfret, and black pomfret. Golden pomfret (*Trachinotus ovatus*) is a commercially important marine fish that primarily inhabits tropical and temperate regions [2]. Owing to its low number of intermuscular bones and tasty flavor, golden pomfret is highly appreciated by diners [3]. Fermented pomfret is produced via natural fermentation based on the interaction of functional and non-functional microbial communities derived from the processing environment and raw materials [4,5]. During fermentation, the microbiota and its enzyme systems contribute to the hydrolysis of lipids and proteins, facilitating the formation of flavor compounds or flavor precursors in fermented foods [6]. Esters are an indispensable component of the unique flavors of fermented foods, and they impart diverse fruit flavors [7]. Esters are primarily synthesized by microorganisms. A wide-range of organic acids and alcohols are generated via microbial metabolism, which then react to generate a variety of flavored esters in reactions catalyzed by carboxylate hydrolase enzymes, such as microbial lipases and esterases [8]. Studies have demonstrated the ability of bacteria [9,10], mycobacteria [11], and yeasts [12] to synthesize flavor esters in various fermented foods and shown that these organisms play important and different roles in flavor synthesis. Indeed, the ester synthesis properties of these microorganisms are dependent on the nature of the functional enzymes they produce.

Carboxylester hydrolases, such as lipases (triacylglycerol acylhydrolases, EC 3.1.1.3) and esterases (carboxylester hydrolases, EC 3.1.1.1), are a group of multifunctional enzymes that catalyze ester synthesis, ester hydrolysis, and ester exchange reactions and are considered promising biocatalysts in biotechnology [13,14]. Given the plurality of traditional fermented foods and the diversity of microorganisms, numerous microbial ester synthases have been identified, cloned, and characterized. In addition, several culturable microbial species could be promising sources of new carboxylate hydrolases [15]. Furthermore, some studies indicate that the application of indigenous strains obtained from specific fermented foods can improve the flavor of foods, as well as ensuring safety and environmental adaptability [9,16]. Traditional fish fermentation processes are often restricted by the level of technology, the processing conditions, and other factors, resulting in a prolonged fermentation period and instability in flavor. Hence, it is important to screen for indigenous microorganisms with high ester synthase activity in traditional fermented fish products and apply them in the preparation and flavor enhancement of fermented fish products.

Genomics, as a branch of biology, includes several sub-disciplines, such as comparative genomics, functional genomics, and macrogenomics. Information about the structure, function, and evolution of a species can be acquired by exploring its genomic features with the support of bioinformatic and computational tools [17]. More importantly, the genes encoding specific biosynthetic enzymes can be excavated from the genome sequence and modified by altering their physical properties, while preserving the necessary coding information, in biocatalytic applications [18,19]. Tepkasikul et al. [20] identified genes responsible for histamine degradation in a halophilic bacterium through whole-gene sequencing. Subsequently, they analyzed and altered the factors influencing enzyme activity, thereby enhancing the histamine degrading activity of the bacterium. Therefore, whole-genome sequencing of a target strain is essential to expand the scope of important enzyme excavations.

In this study, we aimed to identify strains with specific ester synthase activities in traditional fermented pomfret using a combination of gel diffusion screening methods with lipid substrates and complete-genome sequencing. We performed a comprehensive analysis of the genomic features of the selected strains using multiple databases which we mined for functional genes related to ester synthesis. In addition, the homology between functional enzymes and their structural features was analyzed using bioinformatics techniques and databases. Our analysis can facilitate the discovery of new microbial ester synthases with potential applications in the food industry. However, *Acinetobacter venetianus*, which we screened, may cause red leg disease in freshwater-farmed white shrimp [21]; thus, this strain needs to be further studied.

## 2. Materials and Methods

### 2.1. Screening and Identification of Ester Synthase-Producing Bacteria

#### 2.1.1. Isolation and Morphological Identification of Ester Synthase-Producing Microorganisms

Traditional fermented pomfret meat (25 g) was homogenized in a sterile bag with 225 mL of sterile saline. Then, 1 mL of the homogenate was inoculated into 100 mL of enrichment liquid medium (tryptone 1.5 g/100 mL, soy peptone 0.5 g/100 mL, NaCl 0.5 g/100 mL, olive oil 1.0 g/100 mL; pH 7.2 ± 0.2; sterilized at 121 °C for 15 min) and incubated at 30 °C with 180 rpm shaking for 1–2 d until the liquid medium turned turbid. The enriched bacterial broth was serially diluted (10^−2^ to 10^−7^) and spread on the surface of triglyceride agar medium (tryptone 1.0 g/100 mL, yeast extract 0.5 g/100 mL, NaCl 1.0 g/100 mL, agar 1.5 g/100 mL, triglyceride 0.2 mL/100 mL; pH 7.2; sterilized at 121 °C for 15 min), and the formulation of the triglyceride agar medium was slightly modified following Ardö (2006) [22]. The plates were incubated in a constant temperature incubator at 30 °C for 2 days, and individual strains with large clear circles of about 2 mm around them (indicating the hydrolysis of triglyceride) were selected for isolation and preservation. The selected strains were streaked on PDA agar medium and grown at 30 °C for 3–4 days. Sensory evaluation was performed using a sniffing test to further select the strains that contributed to the distinctive ester aroma, alcohol aroma, or other aromatic odors of the plates [23]. This process was carried out by four teachers and three students. Three parallels were made for each sample, and uninoculated PDA was used as a control.

#### 2.1.2. Determination of Enzyme Activity and Total Ester Content

The selected bacterial strains were activated by growing them in seed solution medium (glucose 1.0 g/100 mL, peptone 0.5 g/100 mL, K_2_HPO_4_ 0.05 g/100 mL, MgSO_4_ 0.05 g/100 mL; pH 7.2; sterilized at 121 °C for 15 min), then inoculated into 20 mL of PDA liquid medium with 2% inoculum and cultivated at 30 °C with 180 rpm shaking for 72 h. The total ester content in the fermentation broth was determined using the saponification reflux method according to the Chinese national standard (GB/T 10345-2007 “Analytical Method for White Wine”) [24].

Briefly, 4 mL of fermentation broth was added to a 150 mL Erlenmeyer flask and diluted 10 times with ultrapure water. Thereafter, two drops of phenolphthalein indicator were added, followed by titration with 0.1 mol/L NaOH until the color was faint red (not in excess), and then 25 mL of 0.1 mol/L NaOH was added. The mixture was saponified by refluxing in a boiling water bath for 0.5 h, cooled to room temperature, and immediately titrated with 0.1 mol/L HCl until the red color just disappeared.

The total ester content (in ethyl acetate, g/100 mL) was calculated as follows [24]:

$$\mathrm{Total}\;\mathrm{ester}=\frac{25N_{1}-N_{2}\times V_{1}}{1000}\times 88.12\times 25$$

where *N*_1_ is the concentration of NaOH; *N*_2_ is the concentration of HCl; *V*_1_ is the volume of HCl required for titration; and 88.12 is the molar mass of ethyl acetate (g/mol).

The strains with high ester production ability were activated and inoculated (2% *v*/*v*) into fermentation medium (peptone 1.0 g/100 mL, yeast powder 0.5 g/100 mL, olive oil–polyvinyl alcohol emulsion 12 mL/100 mL, NaCl 0.25 g/100 mL, (NH_4_)_2_SO_4_ 0.2 g/100 mL, K_2_HPO_4_ 0.1 g/100 mL; pH 7.0–7.5; sterilized at 121 °C for 15 min), then incubated for 48 h at 30 °C under 180 rpm shaking. To determine the enzyme activity, the crude enzyme solution was obtained by centrifuging the fermentation broth at 9000× *g* for 4 min at 4 °C. Thereafter, 380 μL of 0.1 mol/L Tris-HCl (pH 8.0) and 100 μL of enzyme solution were added to a 2 mL Eppendorf tube and incubated in a constant temperature water bath at 35 °C for 1–2 min, followed by the addition of 0.036 mol/L p-nitrophenyl phosphate (pNPP) solution (in 3:1 isopropanol/dimethyl sulfoxide) for 5 min. The reaction was terminated by adding an equal volume of SDS solution (18.25 mM/L, with 1.25 M/L of glycine and 0.13 M/L of Tris), and the absorbance of the solution was measured at 405 nm. The enzyme solution was replaced with buffer in the control, with all other conditions kept constant. One unit of enzyme activity (U) was calculated as the quantity of enzyme required to catalyze the hydrolysis of the substrate pNPP to produce 1 μmol p-nitrophenol in 1 min. The total ester content and enzyme activity assays were repeated three times.

#### 2.1.3. Morphological Observation and Identification of Ester Synthase-Producing Microorganisms

##### Morphological Observation of Ester Synthase-Producing Microorganisms

The target strains were morphologically observed using scanning electron microscopy following the method described by M. Maruthupandy et al. [25]. Briefly, the strain with high enzyme activity was inoculated in LB liquid medium and incubated at 30 °C for 24–48 h. Samples were centrifuged at 8000 rpm for 10 min and the supernatant was abandoned. The precipitate was collected and fixed with 50 μL of 2.5% glutaraldehyde at 4 °C for 4 h, then subjected to three washes with 0.1 M phosphate buffer solution (PBS, pH 7.0). Next, the bacterias were eluted sequentially with different gradients of ethanol (30, 50, 25, 70, 85, 90%, and 100%) for 10 min each, except for 100% ethanol, which was eluted twice. After dehydration, the samples were centrifuged at 4 °C for 10 min (10,000 r/min) and the ethanol was replaced with isoamyl acetate twice (20 min/time). The samples were frozen at −20 °C, −40 °C, and −80 °C for 12 h, after which the samples were lyophilized and observed under SEM using an accelerating voltage of 10 kV.

##### Identification of Ester Synthase-Producing Bacteria

DNA was extracted from the selected strains using a bacterial genome extraction kit (Tianjin Biochemical Technology Co., Ltd., Tianjin, China), and 16S rRNA genes were amplified using PCR with the primers 27F (5′ AGAGTTTGATCCTGGCTCAG 3′) and 1492R (5′ TACGGCTACCTTGTTACGACTT 3′). The PCR products were analyzed using 1% agarose gel electrophoresis and visualized under UV light. Sequencing of the PCR products was undertaken by Liuhe Huada Gene Technology Ltd. (Beijing, China). The 16S rRNA sequences were aligned with homologous sequences from the NCBI database. MEGA version 5.05 (Center for Evolutionary Medicine and Informatics, Institute of Biodesign, Arizona State University, Phoenix, AZ, USA) was used to construct the corresponding phylogenetic tree.

### 2.2. Growth Characteristics and Ester Synthase Activity of SCSMX-3

SCSMX-3 was inoculated into seed solution medium at 2% inoculum to explore the effects of temperature (20 °C, 25 °C, 30 °C, 35 °C, and 40 °C), pH (5, 6, 7, 8, and 9), and salinity (0%, 2%, 4%, 6%, 8%, and 10% NaCl) on its growth. OD of the samples at 600 nm was measured after culturing for 24 and 48 h. The activated strain was then inoculated (2% *v/v*) in the fermentation medium to investigate the effects of fermentation temperature, pH, and salinity on the ester synthase activity. These experiments were repeated three times.

### 2.3. Whole Genome Sequencing of SCSMX-3

Genomic DNA extraction was performed according to the instructions of the Wizard^®^ Genomic DNA Purification Kit (Promega, Madison, WI, USA). The purified genomic DNA was quantified using a TBS-380 fluorometer (Turner BioSystems Inc., Sunnyvale, CA, USA). The whole genome of SCSMX-3 was sequenced using a combination of the PacBio RS II Single Molecule Real Time and Illumina sequencing platforms. To ensure high DNA quality for subsequent experiments, the genomic DNA concentration (OD260/280 = 1.8–2.0, total DNA ≥ 1 μg, concentration ≥ 20 ng/μL) was assayed using a NanoDrop2500 when sequenced with PacBio. Illumina sequencing libraries were constructed according to the method described by Aziz et al. (2022) [26]. The assembly and optimization of the genome sequence were implemented using SOAPdenovo2 (http://soap.genomics.org.cn/, accessed on 24 December 2021) [27] and GapCloser. PlasFlow software [28] (https://github.com/smaegol/PlasFlow, accessed on 25 December 2021) was used to find the plasmids in the bacterial genome assembly results. After obtaining the sequences of the plasmids, the plasmids were annotated using the BLAST (https://blast.ncbi.nlm.nih.gov/Blast.cgi, accessed on 25 December 2021) and PLSDB databases (https://ccb-microbe.cs.uni-saarland.de/plsdb/, accessed on 25 December 2021).

### 2.4. Genome Composition Prediction and Gene Function Annotation

Glimmer (http://ccb.jhu.edu/software/glimmer/index.shtml, accessed on 26 December 2021) was used to predict the CDS in the genome [29]. tRNAscan-SE v2.0 (http://trna.ucsc.edu/software/, accessed on 26 December 2021) and Barrnap (https://github.com/tseemann/barrnap, accessed on 26 December 2021) were used to identify tRNA and rRNA, respectively [30]. sRNA annotations were achieved with Infernal software (http://eddylab.org/infernal/, accessed on 26 December 2021) and the Rfam database (https://rfam.xfam.org/, accessed on 26 December 2021). In addition, CRISPR-Cas structures were predicted using MinCED (http://www.room220.com/crt, accessed on 26 December 2021). Finally, the following four databases were used for the annotation of the gene functions: NR (ftp://ftp.ncbi.nlm.nih.gov/blast/db/, accessed on 28 December 2021), the Evolutionary Genealogy of Genes: Non-supervised Orthologous Groups (EggNOG) Database (Version 4.5.1; http://eggnogdb.embl.de/#/app/home, accessed on 28 December 2021), GO (Version 2.5; https://www.blast2go.com/, accessed on 28 December 2021), and KEGG (latest version; http://www.genome.jp/kegg/, accessed on 28 December 2021) [31].

### 2.5. DNA and Protein Sequence Analysis

Sequence alignment was accomplished using Clustal X software [32]. ExPASy online software was used to analyze the physicochemical properties of lipases, such as molecular weight, isoelectric point (PI), and hydrophobicity [33]. Protein secondary structures, such as alpha helix and beta fold, were predicted using NPS@ (http://npsa-bpil.ibcp.fr, accessed on 28 March 2022), which is the “protein part” of the Pôle Bio-Informatique Lyonnais (PBIL). Three-dimensional modeling of lipases was implemented using SwissModel software [34]. Sequence-based prediction of lipase function and superfamily were performed using the Lipase Engineering Database (LED) (http://www.led.uni-stuttgart.de/, accessed on 26 March 2022) [35].

### 2.6. Statistical Analyses

Results are expressed as means plus standard deviation (mean ± SD). Statistical data were analyzed using one-way analysis of variance (ANOVA) with SPSS 22.0 software (SPSS Inc., Chicago, IL, USA). Graphs were constructed using OriginPro 2021. The level of significance was set at *p* < 0.05.

## 3. Results and Discussion

### 3.1. Screening and Identification of Ester Synthase-Producing Strains

**Figure 1:**
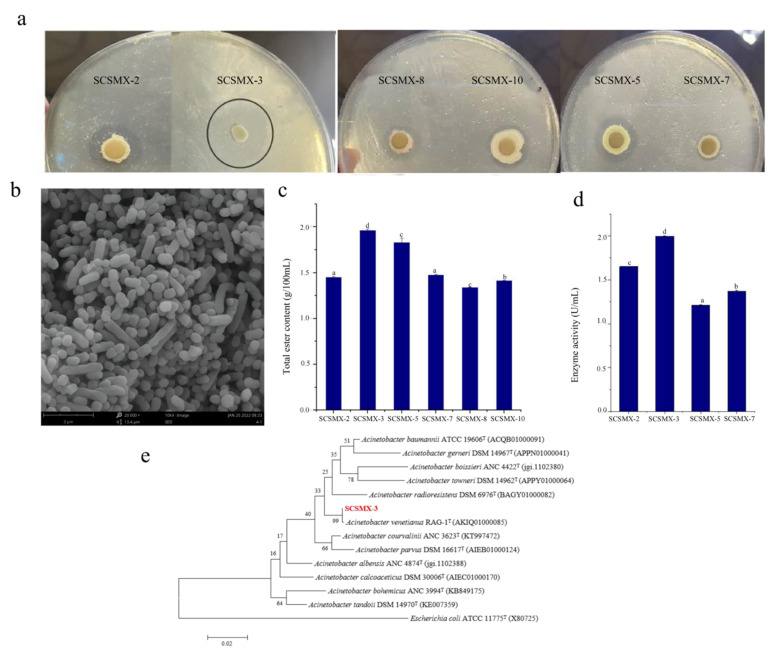
(**a**) Strains exhibiting lipid hydrolysis zones on triglyceride agar plates. (**b**) Electron micrographs of strain SCSMX-3. (**c**) Total ester content produced by SCSMX-2, SCSMX-3, SCSMX-5, SCSMX-7, SCSMX-8, and SCSMX-10. (**d**) Ester synthase activity of SCSMX-2, SCSMX-3, SCSMX-5, and SCSMX-7. (**e**) Phylogenetic tree of SCSMX-3. Different letters in the subfigures indicate significant differences (*p* < 0.05).

Bacterial strains isolated from traditional pomfret were subjected to preliminary screening for their lipid degradation ability in a triglyceride medium by measuring the hydrolysis zone size (Figure 1a). Next, these strains were cultivated on potato dextrose agar (PDA) medium for 72 h, and six strains (SCSMX-2, SCSMX-3, SCSMX-5, SCSMX-7, SCSMX-8, and SCSMX-1) were identified using the sniffing method, which enables detection of the emission of distinct esters, alcohols, or other aromas on PDA plates. The strength of the aroma produced can be determined by measuring the total amount of esters produced in fermented foods by ester synthase-producing strains. Therefore, the saponification reflux method was used to evaluate the total ester content in the fermentation broth [23]. SCSMX-3 produced the highest amount of esters (1.96 g/100 mL total esters) after 72 h of fermentation among the tested strains (Figure 1c). Next, we determined the enzyme activity of the top four strains in terms of ester production. SCSMX-3 showed the highest ester synthase activity of 1.995 U/mL (Figure 1d) and, therefore, it was selected for the subsequent experiments. Electron microscopy (Figure 1b) results revealed that the purified strain was rod-shaped. Furthermore, the 16s rRNA gene of SCSMX-3 was sequenced and homologous sequences were searched for using the BLAST program of the National Center for Biotechnology Information (NCBI) database. The results indicated that the 16S rRNA gene sequence of SCXMX-3 was 100% identical to that of *Acinetobacter venetianus*. Phylogenetic analysis further suggested that SCSMX-3 was most similar to *A. venetianus* RAG-1 (Figure 1e). This strain has been demonstrated to effectively degrade crude oil and exhibit tolerance to a wide range of environmental conditions [36]. Thus, on the basis of its phenotypic features and molecular identification, SCSMX-3 was identified as *A. venetianus* and deposited at the China Microbial Strain Conservation Center under CGMCC number 23774.

### 3.2. Growth Properties of SCSMX-3

**Figure 2:**
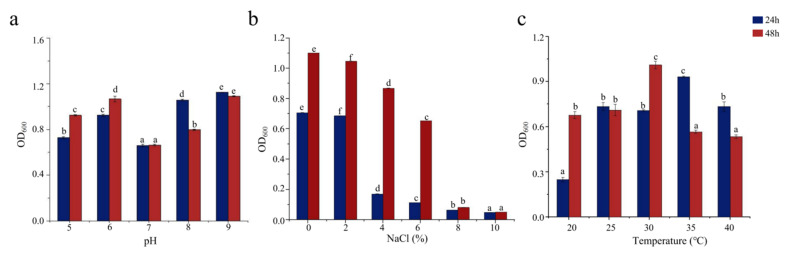
Effects of (**a**) pH, (**b**) salinity, and (**c**) temperature on the growth of SCSMX-3. Different letters in the subfigures indicate significant differences (*p* < 0.05).

Bacterial growth is influenced by pH, salinity, and temperature and is inhibited outside the optimal ranges of these conditions for each bacterium. pH affects the absorption of external nutrients by bacteria. As shown in Figure 2a, SCSMX-3 grew well at pH 6 and in the range from 8 to 9 but showed low growth at pH 7. Therefore, the strain may be alkali-tolerant. SCSMX-3 displayed variable salt tolerance at different incubation times. At 24 h, high cell density was observed under 0–2% NaCl, with growth inhibition at higher salinity (4% NaCl). Incubation for another 24 h resulted in considerably better growth under 0–6% NaCl than at 24 h, indicating an adaptation of the strain to the saline conditions. Higher salt concentrations (8–10% NaCl) severely inhibited growth (Figure 2b). SCSMX-3 also grew well at 20–40 °C, with the maximum cell density at 30 °C, indicating a slight tolerance to high temperature (Figure 2c).

### 3.3. Characterization of the Ester Synthase Activity of SCSMX-3

**Figure 3:**
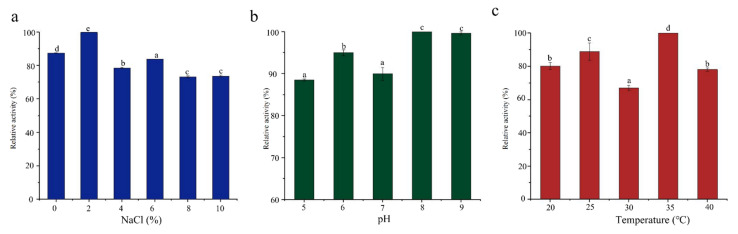
Effect of (**a**) salinity, (**b**) pH, and (**c**) temperature on the ester synthase activity of SCSMX-3 in the fermentation broth. Different letters in the subfigures indicate significant differences (*p* < 0.05).

Salinity, pH, and temperature directly affect the growth of bacteria, thereby affecting enzyme production [37,38]. SCSMX-3 presented maximum ester synthase activity under 2% salinity when fermented for 48 h (Figure 3). The enzyme activity decreased with the increase in salinity (4–10% NaCl) but still presented more than 84% enzyme activity. Typically, the optimal pH for bacterial lipase activity is in the alkaline or neutral range [39]. The ester synthase from SCSMX-3 was observed to be active between pH 5.0 and 9.0, with the maximum enzyme activity at pH 8.0, demonstrating that this enzyme may be a moderately alkaliphilic lipase. The enzyme also exhibited high activity in the 20–40 °C temperature range, with the maximum activity at 35 °C. Interestingly, the maximum activity for the ester synthase produced by *A. venetianus* SCSMX-3 was at the same optimal pH and temperature as those of the ester synthase produced by *Streptomyces* sp. Al-Dhabi-49 [40]. Some studies have revealed that fermentation at the appropriate temperature and pH can not only control the growth of undesirable and harmful bacteria in fermented foods but also enhance the flavor quality of fermented foods [41,42].

### 3.4. Complete-Genome Sequencing and Information Analysis

**Figure 4:**
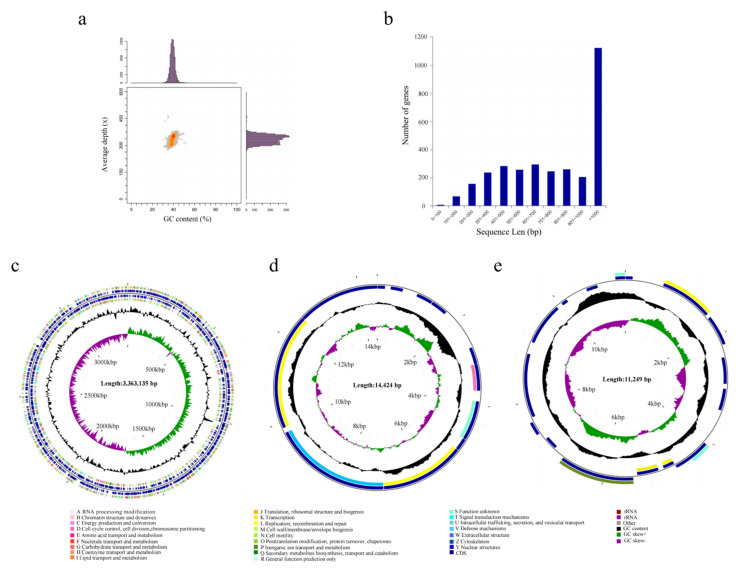
(**a**) GC-depth analysis of SCSMX-3 genome. (**b**) Gene length distribution. (**c**) CGView chromosome circle group map. (**d**) CGView plasmid A circle group map. (**e**) CGView plasmid B circle group map. Note: The first and fourth circles from outside to inside are CDSs on the forward strand and reverse strand, and different colors indicate various COG functional classifications; the second and third circles are CDSs, tRNA, and rRNA on the forward strand and reverse strand, respectively; the fifth circle is the G+C content, and the outward part shows that the G+C content of the region is higher than the average G+C content of the whole genome, while the inward part shows that the G+C content of the region is lower than the average G+C content of the whole genome; the higher the peak, the bigger the gap with the average G+C content; the sixth circle is the G+C-Skew value.

To reveal the functional genes associated with ester metabolism in *A. venetianus* SCSMX-3, its whole genome was sequenced and analyzed using the Illumina HiSeq and PacBio sequencing platforms. A total of 261,387 reads were obtained after quality clipping of the raw data, with the longest and average read lengths of 274,729 bp and 11,726.81 bp, respectively. The statistical plot of 17-Kmer displayed only one main peak without trailing after the main peak, demonstrating that there are no significant repetitive sequences in the genome (Figure S1). An analysis of the GC depth revealed that most of the points are concentrated in a relatively narrow range, indicating a relatively low possibility of sample contamination (Figure 4a). The whole genome of SCSMX-3 consists of a circular chromosome 336,313 bp long, with 39.18% G+C content. In addition, it contains two circular plasmids, plasmid A (14,424 bp, 33.73% G+C) and plasmid B (11,249 bp, 36.15% G+C). The coding sequences (CDSs) in the genome were predicted using the Glimmer, GeneMarkS, and Prodigal software packages (Table 1 and Table S1). A total of 3150 CDSs were acquired, with an average G+C content of 40.09%, 73 tRNAs, 18 rRNAs, and 17 sRNA operons. Most of the genes identified in the CDSs were greater than 1000 bp in length (Figure 4b). The circular chromosome was predicted to include 3212 open reading frames (ORFs), and 12 and 17 ORFs were detected in plasmid A and plasmid B, respectively. The circular genome map of the chromosome and two plasmids of SCSMX-3 was drawn using CGView, showing the gene distribution profile from a macroscopic perspective (Figure 4c–e).

### 3.5. CRISPR Prediction

The CRISPR system is a cluster of regularly spaced short palindromic repeats existing in prokaryotic genomes, including bacteria and archaea. This system allows the storage of adaptive immune genetic information, which provides acquired immunity to prokaryotes [43]. Through genome-wide prediction using Minced [44], six CRISPR structures were identified in *A. venetianus* SCSMX-3 (Table 2). The presence of CRISPR structures may improve the genome stabilization of SCSMX-3, leading to increased environmental adaptability. Application of CRISPR/Cas9 or CRISPR/Cpf1 systems constructed using gene editing techniques has been reported for the genetic engineering of *Aspergillus oryzae* [45], the ascomycete fungus *Penicillium subrubescens* [46], and other filamentous fungi, especially for mutagenesis and gene deletion/integration genetic engineering, which is essential to further improve the functional characterization of the strains and their genes. Consequently, the CRISPR structures embedded in *A. venetianus* SCSMX-3 have the potential to contribute to genetic modification for the generation of functional strains.

### 3.6. Genome Function Annotation

**Figure 5:**
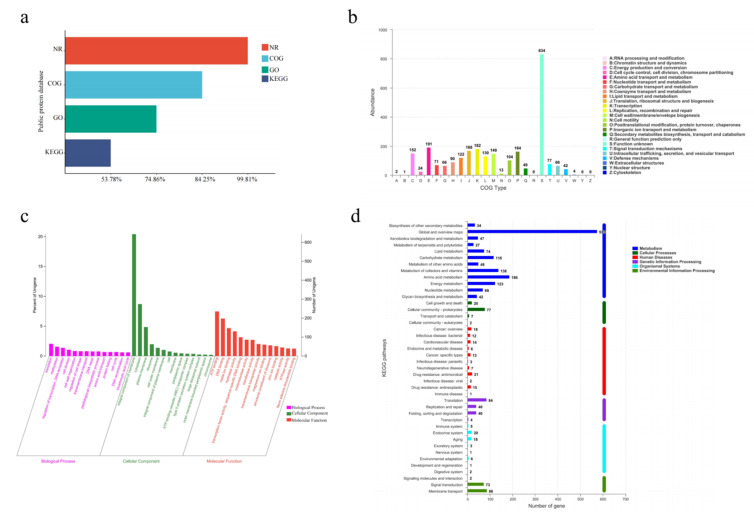
(**a**) Functional annotation and percentages of SCSMX-3 genes in public databases. (**b**) COG annotation of SCSMX-3 genes. (**c**) GO annotation of SCSMX-3 genes. (**d**) KEGG annotation of SCSMX-3 genes.

To further explore the functional diversity of proteins encoded by different genes, the predicted amino acid sequences of *A. venetianus* SCSMX-3 were annotated using the NCBI Non-Redundant Protein Database (NR) and the Gene Ontology (GO), Kyoto Encyclopedia of Genes and Genome (KEGG), and COG databases (descriptions provided in the Materials and Methods section). Almost all genes were annotated in the NR database (3144/99.81%), followed by the COG (2654/84.25%) and KEGG (1694/53.78%) databases (Figure 5a).

The COG database can classify gene families of predicted proteins to identify the function of the target sequence. There were 2654 predicted proteins, which were sorted into 22 categories upon mapping to the COG database (Figure 5b). Although most genes were annotated to proteins of unknown functions (n = 834), SCSMX-3 was found to be enriched in functional genes associated with the transport and metabolism of nutrients, such as proteins and lipids, including amino acid transport and metabolism (n = 191); translation, ribosome structure, and biogenesis (n = 168); and lipid transport and metabolism (n = 122). The findings indicate that this strain is highly capable of synthesizing flavor compounds. Furthermore, the strain is abundant in genes associated with transcription (n = 182); energy production and transformation (n = 152); cell wall/membrane/envelope biogenesis (n = 149); and replication, recombination, and repair (n = 130), indicated a higher growth viability.

While the GO database clusters genes with the same function, the KEGG database systematically analyzes the functional information of the genome. Using these databases simultaneously can contribute to a better understanding of gene function [47]. Among the 2358 genes annotated in the GO database, 1160, 1912, and 1113 genes were related to cell composition, molecular function, and biological processes, respectively. A significant number of genes relevant to molecular function were involved in ATP binding and DNA binding processes. A significant number of genes associated with cell composition were related to membrane components. Genes related to translation, transcriptional regulation, and cell division were abundant in the biological processes group (Figure 5c). These findings indicate that SCSMX-3 may possess excellent proliferative ability.

Metabolic pathway predictions using the KEGG database revealed that 1479, 168, 161, 112, 106, and 56 genes were associated with metabolism, genetic information processing, environmental information processing, human diseases, cellular processes, and biological systems, respectively. The genes involved in metabolism were distributed in the global and overview map (575), followed by amino acid metabolism (186), carbohydrate metabolism (116), metabolism of cofactors and vitamins (138), energy metabolism (123), and lipid metabolism (74). Interestingly, the genes involved in environmental information processing at the highest percentage were membrane transport genes. In the secondary classification of genetic information processing, the highest number of genes were involved in translation (Figure 5d). These results indicate that SCSMX-3 has a strong capacity for energy metabolism, flavor substance synthesis, and substance transport [48].

### 3.7. Analysis of Genes Related to Lipid Metabolism

**Figure 6:**
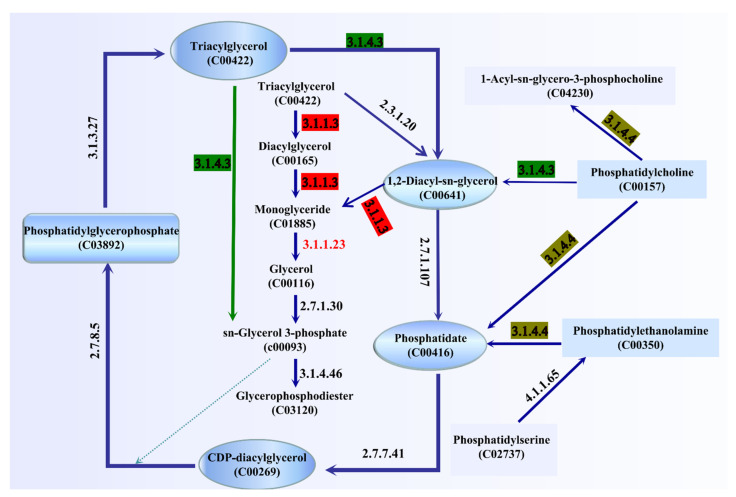
Pathway analysis of enzymes associated with lipid metabolism. The enzymes involved are indicated by their EC numbers: EC 3.1.1.3: triacylglycerol lipase; EC 2.7.1.30: glycerol kinase; EC 3.1.1.23: acylglycerol lipase; EC 3.1.4.3: phospholipase C; EC 3.1.4.46: glycerophosphodiester phosphodiesterase; EC 3.1.4.4: phospholipase D; EC 4.1.1.65: phosphatidylserine decarboxylase; EC 2.7.1.107: ATP:1,2-diacyl-sn-glycerol 3-phosphotransferase; EC 2.7.7.41: CTP: phosphatidate cytidylyltransferase; EC 2.7.8.5: CDP-diacylglycerol:sn-glycerol-3-phosphate 1-(3-sn-phosphatidyl) transferase; EC 3.1.3.27: phosphatidylglycerophosphate phosphohydrolase; EC2.3.1.20: diacylglycerol O-acyltransferase. The green arrow indicates that sn-glycerol-3-phosphate can be generated directly by triacylglycerol.

The ester synthase activity of *A. venetianus* SCSMX-3 was experimentally proven in this study. The whole-genome sequence of SCSMX-3 was therefore analyzed using the NR database, which identified a total of 42 genes linked to lipid metabolism. Among these, seven, three, and two genes were identified as encoding acyl-CoA thioesterase, triacylglycerol lipase, and esterase, respectively (Table S2). We mapped the 42 genes to the KEGG database, and the potential pathways of lipid metabolism in SCSMX-3 are proposed (Figure 6). Triglycerides (TAG) can be hydrolyzed to monoglycerides (MAG) by the action of triacylglycerol lipase (EC 3.1.1.3). However, the genes encoding glycerol-ester acyl hydrolase (EC 3.1.1.23) are hormone-sensitive lipase (EC 3.1.1.79), which are responsible for the production of glycerol from MAG, are not present in this strain, suggesting that SCSMX-3 may not be capable of fully hydrolyzing triglycerides. Genes encoding phosphatidylglycerophosphate phosphohydrolase (EC 3.1.3.27), phospholipase C (PLC, EC 3.1.4.3), ATP:1,2-diacyl-sn-glycerol 3-phosphotransferase (EC 2.7.1.107), CTP:phosphatidate cytidylyltransferase (EC 2.7.7.41), and CDP-diacylglycerol:sn-glycerol-3-phosphate1-(3-sn-phosphatidyl)transferase (EC 2.7.8.5) were identified in SCSMX-3, which are involved in the degradation and synthesis of phosphatidylglycerophosphate. Phosphatidylglycerol phosphatase, a membrane-associated enzyme, catalyzes the generation of multi-functional phosphatidylglycerol from phosphatidylglycerol phosphate, thereby facilitating the synthesis of a variety of surface molecules [49]. In addition to the synthesis of phosphatidylglycerol, PLC is responsible for the hydrolysis of phospholipids and generates diacylglycerol (DG) and organophosphates. Importantly, PLCs from microbial sources are suitable for industrial application because of the simplicity of culturing these microbes and their adaptability to the environment [50,51]. Thus, SCSMX-3 provides the opportunity to widen the scope of PLCs of microbial origin. Additionally, the enzyme-coding genes correlated with the synthesis and hydrolysis of phosphatidylethanolamine, the product of which, phosphatidic acid (PA), is an important intermediate in the biosynthesis of all membrane glycerophospholipids and storage lipid-TAG, are also present in SCSMX-3. Through the action of CTP:phosphatidate cytidylyltransferase, PA can be transformed into CDP-diacylglycerol (CDP-DG), a lipid nucleotide intermediate that serves as a common precursor for the ab initio biosynthesis of phosphatidylinositol and cardiolipin [52]. The gene encoding phosphatidylcholine 2-acylhydrolase (EC 3.1.1.4), which catalyzes the hydrolysis of the sn-2 position of phospholipids to produce lysophosphatidylcholine and free fatty acids, was also identified in SCSMX-3 [53]. This enzyme is widely applied in the preparation of dairy and bakery products [54].

### 3.8. Genetic Informatic Analysis of the Triacylglycerol Lipase of A. venetianus SCSMX-3

**Figure 7:**
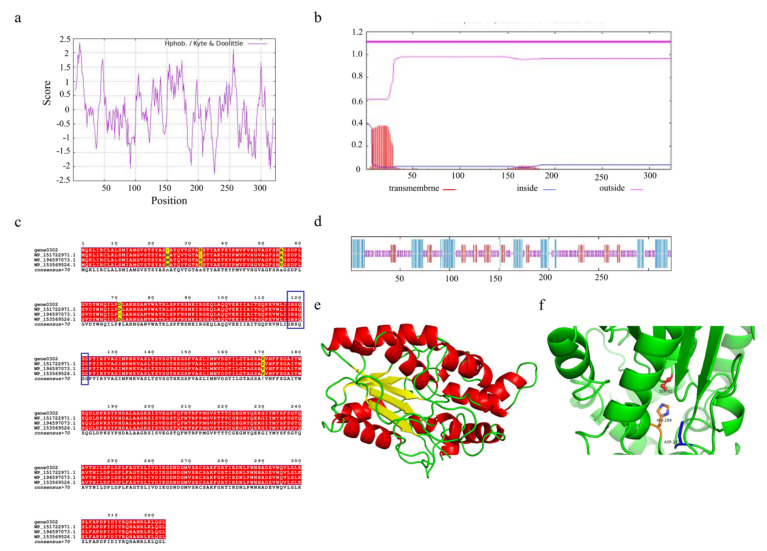
(**a**) Hydrophobicity profile of SCSlip-1 lipase. (**b**) Predicted transmembrane structure of SCSlip-1. (**c**) Multiple amino acid sequence alignment of SCSlip-1 and some other microbial lipases. (**d**) Secondary structure information of SCSlip-1. (**e**,**f**) Tertiary structure and catalytic site distribution of SCSlip-1.

Understanding the structural features of enzymes is essential to design or engineer them for industrial applications. The triacylglycerol lipase encoded by gene0302 and gene1402 consists of 323 and 127 amino acids, respectively. Due to the higher number of amino acids encoded by gene0302, we selected this lipase for its potential physicochemical properties. The triacylglycerol lipase encoded by gene0302, named SCSlip-1, has 4840 atoms (molecular formula C_1532_H_2409_N_423_O_464_S_12_) and a molecular weight of 34.56 kD, isoelectric point (pI) of 8.36, and 25 and 23 positively and negatively charged residues, respectively. The instability index is 24.57 (<40), which indicates that the protein conformation is relatively stable. ProtScale analysis of SCSlip-1 revealed a total average hydrophobicity score of −0.0741, with the strongest hydrophobicity score of 2.356 for amino acid 11 and −2.267 for amino acid 226. As the average hydrophobicity of the protein is in a negative state, the lipase was determined to be hydrophilic (Figure 7a). No transmembrane region was observed, and all 323 amino acids were outside the membrane (Figure 7b).

To further explore the characteristics of SCSlip-1, we analyzed the conserved structural domain of SCSlip-1 and performed multiple sequence alignment with other lipases. The results showed that the enzyme encoded by gene0302 belonged to the alpha/beta hydrolases superfamily and was annotated as a triacylglycerol lipase, sharing the highest homology (99.38%) with the triacylglycerol lipase of *Acinetobacter junii* (WP_153569526.1). In addition, the comparison revealed the presence of a conserved serine-containing pentapeptide GHSQG (Figure 7c, blue box), revealing serine as a catalytic residue in a binding site for lipase substrates [55]. Prediction of the secondary structures of the lipase can contribute to understanding the relationship between lipase function and its amino acid sequence, as well as the prediction of its tertiary structure [56,57]. Therefore, the secondary structure of SCSlip-1 was predicted using the DSC, HNNC, MLRC, PHD, and Predator methods in NPS@. Although the measurements (Table S3) from different prediction methods exhibited a certain variability, the consistent prediction results revealed a final percentage of 24.46% α-helix, 15.17% extended strand, and 56.04% random coil, suggesting that α-helix and random coil are the main components of SCSlip-1 (Figure 7d). A comparison with the Lipase Engineering Database (LED) revealed that SCSlip-1 belonged to class GX, abH15.02 (Burkholderia cepacian lipase) homolog of the abH15 superfamily. The GX category includes a superfamily of lipases in which G is the conserved glycine and X is the amino acid that forms the oxygen anion, showing a preference for the hydrolysis of medium and long carbon chain substrates [35]. Moreover, the catalytic triad residues of SCSlip-1 were identified as S82, D267, and H259, respectively. Using the Swiss-Model online software, we established the tertiary structure of SCSlip-1 with *Pseudomonas aeruginosa* lipase 1EX9 A as the template (47.28% sequence identity). The results are shown in Figure 7e,f. Our results predicted the tertiary structure of lipase derived from *A. venetianus*; however, the properties of purified lipase and its hydrolysis products require further investigation.

## 4. Conclusions

In conclusion, the *A. venetianus* SCSMX-3 strain was isolated from traditional fermented golden pomfret, and it exhibited low temperature- and low salt-tolerance. It grew well under conditions of up to 6% NaCl in a wide pH range and produced an ester synthase with high catalytic efficiency. Whole-genome sequencing revealed the presence of one circular chromosome and two circular plasmids in this strain. Importantly, a considerable number of genes from the SCSMX-3 genome were found to participate in energy metabolism and flavor substance synthesis, and three genes encoding triacylglycerol lipase and two encoding esterases were identified. The triacylglycerol lipase (SCSlip-1) encoded by gene0302 belongs to the GX class of lipases, which prefers medium and long carbon chain lipid substrates for hydrolysis. Our findings provide a better comprehension of the genome of *A. venetianus* and its metabolic profile associated with lipids, revealing genomic information for the future development of new ester synthases using genetic tools and entailing considerable implications for the improvement of the flavor quality of fermented fish products.

## Figures and Tables

**Table 1:** Gene and non-coding RNA information statistics for the SCSMX-3 genome.

Type	Total Number	Average Length (bp)	Total Length (bp)	GC Content (%)
Gene	3150	943.93	2,973,366	40.09%
tRNA	73	76.835	5609	-
5s_rRNA	6	109	654	-
16s_rRNA	6	1531.833	9191	-
23s_rRNA	6	2888	17,328	-
sRNA	17	132.647	2255	-

**Table 2:** Results of CRISPR prediction for SCSMX-3 genome.

Location	CRISPR ID	Start	End	Duplicate Sequences Average Length (bp)	Duplicate Sequences Average Length (bp)	Spaced Sequences Average Length (bp)
Chromosome	CRISPR1	378,376	378,604	4	31	35
Chromosome	CRISPR2	438,986	440,099	20	31	26
Chromosome	CRISPR3	441,911	442,682	14	31	26
Chromosome	CRISPR4	2,033,076	2,033,818	11	23	49
Chromosome	CRISPR5	2,034,295	2,034,592	4	46	38
Chromosome	CRISPR6	2,038,236	2,039,478	15	28	58

## Data Availability

The data presented in this study are available on request from the corresponding author. The detailed policies could be found by the following link: https://www.mdpi.com/ethics#13.

## Supplementary Materials

The following supporting information can be downloaded at: https://www.mdpi.com/article/10.3390/foods11131954/s1, Figure S1: kmer = 17 Depth-Frequency Distribution; Table S1: General characteristics of *Acinetobacter venezia* SCSMX-3 genome; Table S2: NR data esterase (lipase) annotation information; Table S3: NR data esterase (lipase) annotation information.
