# Supplementary material for: Whole-Genome Sequencing of a Potential Ester-Synthesizing Bacterium Isolated from Fermented Golden Pomfret and Identification of Its Lipase Encoding Genes
Source: Foods. 2022 Jun 30;11(13):1954. doi: 10.3390/foods11131954 (PMC9266206; doi:10.3390/foods11131954)
Supplement: Supplementary file 1 [file foods-11-01954-s001.zip › foods-1732396-supplementary.pdf]

Supplementary Materials:

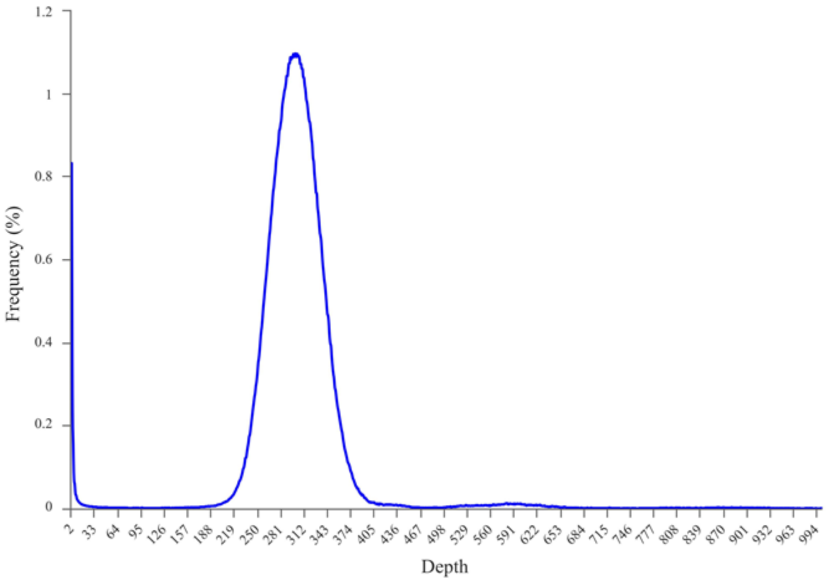

Figure S1. kmer=17 Depth-Frequency Distribution.

Table S1. General characteristics of *Acinetobacter venezia* SCSMX-3 genome.

Characteristic	Chromosome	Plasmid A	Plasmid B
Gene Total Length（bp）	3363135	14425	11249
G+C content（%）	39.18%	33.73%	36.15%
ORFs	3212	12	17
5s_rRNA	6	-	-
16s_rRNA	6	-	-
23s_rRNA	6	-	-
tRNA	74	-	-
A-base content	1026554	4544	3703
T-base content	1018779	5015	3479
G-base content	662299	2225	1887
C-base content	655503	2640	2180
tandem repeat	67	1	1

Table S2. NR data esterase (lipase) annotation information.

Gene ID	Hit-Description	Gene Len	Identity	Evalue	KEGG Name	KEGG Functional Role
gene0072	phosphatidylglycerophosphatase A	173	99.4	4.10E-96	pgpA	Phosphatidylglycerophosphatase（3.1.3.27）
gene0212	imidazole glycerol phosphate synthase subunit HisH	205	100	7.60E-118		
gene0302	triacylglycerol lipase	323	99.1	5.90E-181	lip	Triacylglycerol lipase(3.1.1.3)
gene0370	acyl-CoA thioesterase II	290	99.3	9.00E-165	tesB	Thioester hydrolases（3.1.2.-）
gene0476	phospholipase D family protein	542	99.6	0		
gene0477	metallophosphoesterase	336	100	8.80E-196		
gene0490	acyl-CoA thioesterase	139	99.3	8.10E-71	yciA	Thioester hydrolases（3.1.2.-）
gene0780	Pimeloyl-[acyl-carrier protein] methyl ester esterase	249	90.4	1.40E-121		
gene0820	patatin-like phospholipase family proteinPatatin-like	596	99.8	0		
gene0897	thioesterase family protein	165	99.4	1.00E-88		
gene1060	glycerophosphodiester phosphodiesterase	186	93.5	6.50E-92	glpQ	glycerophosphodiester phosphodiesterase（3.1.4.46）
gene1150	PaaI family thioesterase	144	100	1.70E-76	-	-
gene1263	3-oxoadipate enol-lactonase	261	97.3	3.80E-138	-	-
gene1316	acyl-CoA thioesterase	131	96.2	1.00E-67	ybgC	Thioester hydrolases（3.1.2.-）
gene1358	beta-ketoacyl-ACP synthase III	368	100	1.50E-201	-	-
gene1376	triacylglycerol lipase	411	98.8	1.60E-228	-	-
gene1402	triacylglycerol lipase	127	100	4.30E-63	lip	triacylglycerol lipase（3.1.1.3）
gene1550	phospholipase A	389	99.2	2.00E-223	-	-
gene1569	lipase	239	97.1	3.60E-127	-	-
gene1587	phospholipase D family protein	482	99.2	1.40E-279	-	-
gene1778	acyl-CoA thioesterase	146	100	7.90E-77	ybgC	Thioester hydrolases（3.1.2.-）
gene1941	PaaI family thioesterase	138	99.3	2.30E-70	-	-
gene1968	esterase	420	97.9	3.80E-241	-	-
gene2130	acyl-CoA thioesterase	154	98.1	1.50E-81	ybgC	Thioester hydrolases（3.1.2.-）
gene2138	esterase	200	95	1.10E-105	-	-
gene2298	arylesterase	209	100	7.80E-110	tesA	acyl-CoA thioesterase I（EC:3.1.2.- 3.1.2.2 3.1.1.2 3.1.1.5）
gene2300	Lipase 1 precursor	338	99.7	5.70E-187	-	-
gene2309	diacylglycerol kinase	124	100	1.70E-56	dgkA	diacylglycerol kinase (ATP)（2.7.1.107）
gene2314	metallophosphoesterase	251	98.8	3.90E-148	-	-
gene2320	glycerol kinase GlpK	504	99.8	2.90E-296	-	-
gene2337	acyl-CoA thioesterase	173	96.5	1.70E-89	-	-
gene2370	glycerophosphodiester phosphodiesterase	378	97.9	3.30E-215	glpQ	glycerophosphodiester phosphodiesterase（3.1.4.46）
gene2467	patatin-like phospholipase family proteinpatatin	318	100	1.90E-163	-	-
gene2508	patatin-like phospholipase family proteinpatatin	314	99.4	1.30E-169	-	-
gene2600	glycerophosphodiester phosphodiesterase	239	99.2	4.70E-135	glpQ	glycerophosphodiester phosphodiesterase（3.1.4.46）
gene2617	phospholipase	254	95.7	1.60E-136	-	-
gene2650	beta-ketoacyl-ACP synthase	407	99.8	3.90E-230	-	-
gene2658	acyl-CoA thioesterase	142	100	3.10E-78	ybgC	Thioester hydrolases（3.1.2.-
gene2667	beta-ketoacyl synthase chain length factor	204	100	3.10E-111	plc	phospholipase C（3.1.4.3）
gene2695	sphingomyelin phosphodiesterase	448	99.3	1.30E-255	-	-
gene3145	phospholipase C, phosphocholine-specific	726	99.6	0	-	-

Table S3. NR data esterase (lipase) annotation information.

	Alpha helix	Extended strand	Random coil
DSC	24.46%	13.31%	62.23%
HNNC	28.79%	20.43%	50.77%
MLRC	30.34%	15.48%	54.18%
PHD	33.75%	26.32%	39.94%
Predator	11.76%	20.43%	67.80%
Sec.Cons.	24.46%	15.17%	56.04%
